# Serum biomarkers in patients with hand-arm vibration injury and in controls

**DOI:** 10.1038/s41598-024-52782-1

**Published:** 2024-02-01

**Authors:** Eva Tekavec, Tohr Nilsson, Lars B. Dahlin, Elizabeth Huynh, Anna Axmon, Catarina Nordander, Jakob Riddar, Monica Kåredal

**Affiliations:** 1 Division of Occupational and Environmental Medicine, Department of Laboratory Medicine, Lund University, 221 00 Lund, Sweden; 2 Section of Sustainable Health, Department of Public Health and Clinical Medicine, Umeå University, SE-901 87, Umeå, Sweden; 3 Department of Translational Medicine-Hand Surgery, Lund University, 221 00 Lund, Sweden; 4 Occupational and Environmental Medicine, Region Skåne, 223 63 Lund, Sweden

**Keywords:** Diseases, Health occupations, Medical research, Neurology, Rheumatology, Risk factors

## Abstract

Hand-arm vibration injury is a well-known occupational disorder that affects many workers globally. The diagnosis is based mainly on quantitative psychophysical tests and medical history. Typical manifestations of hand-arm vibration injury entail episodes of finger blanching, Raynaud’s phenomenon (RP) and sensorineural symptoms from affected nerve fibres and mechanoreceptors in the skin. Differences in serum levels of 17 different biomarkers between 92 patients with hand-arm vibration injury and 51 controls were analysed. Patients with hand-arm vibration injury entailing RP and sensorineural manifestations showed elevated levels of biomarkers associated with endothelial injury or dysfunction, inflammation, vaso- or neuroprotective compensatory, or apoptotic mechanisms: intercellular adhesion molecule-1 (ICAM-1), monocyte chemoattractant protein-1 (MCP-1); thrombomodulin (TM), heat shock protein 27 (HSP27); von Willebrand factor, calcitonin gene-related peptide (CGRP) and caspase-3. This study adds important knowledge on pathophysiological mechanisms that can contribute to the implementation of a more objective method for diagnosis of hand-arm vibration injury.

## Background

The clinical assessment of the vascular and sensorineural components of hand-arm vibration injury, diagnosis and grading are based mainly on the subjectively reported medical history and the psychophysical assessments of quantitative sensory tests^1^. An objective method, e.g., measuring serum levels of biomarkers, is therefore desirable. Furthermore, biomarkers could help us understand the pathophysiology and prognosis better. Several pathophysiological mechanisms have been proposed, e.g., localized injury to blood vessels and nerves, as well as systemic inflammatory processes^2–5^.

The vascular component of hand-arm vibration injury entails episodic attacks of vasoconstriction, described as Raynaud’s phenomenon (RP). For correct diagnosis of vibration-induced RP, other conditions causing RP, e.g., connective tissue diseases, have to be ruled out^6^. The pathophysiology of RP has been suggested to include both structural changes and dysfunction of the blood vessel wall with loss of the control of the vascular tone^6^. Various biomarkers related to endothelial cell function and vascular integrity, as well as inflammation, have been suggested as early markers of disease. Elevated levels of von Willebrand factor (vWf) have been shown in individuals with RP who subsequently developed a connective tissue disease^7^. Elevated levels of biomarkers associated with angiogenesis, tissue remodelling, fibrosis and wound repair, i.e., basic-fibroblast growth factor (b-FGF), hepatocyte growth factor (HGF), vascular endothelial growth factor (VEGF) and matrix metalloproteinases (MMP-1 and MMP-12), have been detected in cases of RP caused by a connective tissue disease^8^. It is not clear whether elevated levels of such biomarkers can also be detected in individuals with vibration-induced RP, and if the serum levels resemble those in individuals with primary or with secondary RP due to a connective tissue disease. However, in a study on individuals with vibration injuries, levels of thrombomodulin (TM) were found to be within the range in individuals with RP due to connective tissue disease^9–11^. Studies of blood levels of vWf in vibration-injured individuals have shown varying results^12,13^.

The blood vessel walls are constantly exposed to shear stress and cyclic stretch by hemodynamic forces, physiologically generated by the vascular tone, blood viscosity and cardiac output^14^. Reduced luminal radius size, as an acute effect of vibration exposure, has been shown in a rat tail model^15^, after continuous vibration^4^, and in an experimental setting in fingers of exposed individuals, as well as in the non-exposed hand^16,17^. Endothelial cells have shown to respond to increased mechanical strain associated with mechanical ventilation by promoting inflammation, adhesion, and contractility leading to vascular dysfunction^14^. Intercellular adhesion molecule 1 (ICAM-1) and monocyte chemoattractant protein-1 (MCP-1) are biomarkers that have shown to be upregulated under conditions of stress or injury and enhance the inflammatory response by attracting and facilitating the migration of inflammatory cells from the blood stream into the injured tissue^18^. Changes in cyclic stretch and shear stress to endothelial cells has shown changes in levels of biomarkers associated with angiogenesis, vascular remodelling, adhesion and inflammation (i.e., MMP-1, HGF, VEGF, ICAM-1)^14^. Elevated plasma levels of ICAM-1 have also been shown in vibration injured patients compared to controls^19^. Serum levels of ICAM-1 have been correlated to disease activity in patients with RP due to scleroderma and in fact decreasing levels were observed upon drug-induced vasodilation with a prostaglandin analogue [29]. MCP-1 has to the best of our knowledge not been studied in relation to vibration injury.

The vascular tone is balanced by vasoconstricting and vasodilating factors. Overproduction of endothelin-1 (Et-1), a potent vasoconstrictor, has been found in patients with primary RP^8^. Elevated levels of Et-1 were associated with structural changes in arteries in rat tail exposed to vibration: i.e., vacuole formation in endothelial and smooth muscle cells, disruption of the endothelial cells with discontinuity of the internal elastic membrane, and swelling and cavitation of mitochondria^20^ in individuals with vibration-induced RP varying blood levels of Et-1 have been reported^12,21^.

Calcitonin gene-related peptide (CGRP), a well-known vasodilator, has been studied in patients with RP^22^. Interestingly, neurogenic vasodilation via CGRP has been linked to transient receptor potential ankyrin 1 (TRPA-1)^23^. TRPA-1 acts as a sensor to cold and is associated with cold intolerance and inflammatory pain. In an animal model cold hypersensitivity could be induced in mice by subcutaneous administration of a TRPA-1 agonist, while TRPA-1 antagonist reduced cold hypersensitivity^24^. TPRA-1 expression in serum has not been studied in vibration-injured patients, but, since cold intolerance, a prominent symptom among these patients has been association with the sensorineural component of injury^25^, it is of considerable interest.

The neurosensory component of hand-arm vibration injury entails clinical manifestations from small (Aδ and unmyelinated C) and large (Aβ) sensory nerve fibres, as well as sensory receptors in the skin. Structural changes, such as demyelination and axonal degeneration, have been observed in nerve biopsies from vibration-exposed individuals^26^ and in experimental models^27^, suggesting that certain nerve biomarkers may be elevated. Structural changes may be the mechanism(s) underlying increased susceptibility to nerve entrapment in vibration-exposed individuals, according to light microscopic findings in individuals with diabetes, where a higher prevalence of nerve entrapment has been observed^28,29^. Finger biopsies from patients with vibration injuries have shown Schwann cell activation, an increase in fibroblast cell number, fibrosis, loss of the myelin sheath, and reductions in the elastic membrane and axonal size^2,30–32^. These findings have been confirmed in experimental studies^4,33^. Nerve degeneration induces a variety of biological processes in the affected nerve, including inflammatory response with macrophage recruitment, Schwann cell proliferation and apoptosis with compensatory neuroprotective mechanisms, as seen in various traumatic injuries and in neuropathies. The apoptotic response in Schwann cells, for example, can be measured as increased levels of caspase-3, which balance the proliferative response after a nerve injury. Some vibration-injured patients suffer badly from pain in their hands, interestingly, plasma levels of galanin, an important substance in a variety of functions, including nociception, is increased after traumatic nerve injuries and in individuals after vibration exposure^34^. Furthermore, glial fibrillary acidic protein (GFAP), a proposed marker for axonal damage^35^, has been detected in nerve biopsies in patients with type 2 diabetes and controls^36^, and elevated serum levels of GFAP were found to correlate to reduced nerve action potentials and disease severity in chronic neuropathies^37^. Furthermore, a tendency of decreased levels of myelin basic protein (MBP), a lipid-interacting protein of myelin, and higher levels of GFAP have been observed in nerve biopsies from subjects with type 2 diabetes compared to healthy subjects^36^. GFAP and MBP have, to the best of our knowledge, not been studied in relation to hand-arm vibration injury. Neuronal survival, axonal outgrowth, synaptic plasticity and neurotransmission have been reported to be regulated by neurotrophins, including β-nerve growth factor (β-NGF), brain-derived neurotrophic factor, neurotrophin-3 (NT-3), and neurotrophin-4/5, which bind to tyrosine kinase receptors TrkA, TrkB, and TrkC, and the common neurotrophin receptor, p75NTR^38^. Neurotrophins may act as axonal guidance molecules during nerve regeneration, as well as having neuroprotective properties after injury, and may even stimulate nerve regeneration^38^. Neuroprotection includes activation of heat shock proteins (HSPs) such as HSP27, detected in human nerve biopsies, which act as chaperones to protect nerve structures under stress^36,39^ to preserve nerve function. Hypothetically, HSPs may be upregulated in individuals with hand-arm vibration injuries to protect the nerves and to prevent apoptosis^40^. Hence, biomarkers reflecting endothelial injury or dysfunction, inflammation, nerve injury and tissue remodelling of extracellular matrix were selected for assessment of injury.

The aim of this study was to assess serum levels of several of these biomarkers in individuals with hand-arm vibration injuries to improve our knowledge on pathophysiological mechanisms, to identify objective markers as candidates for accurate and timely diagnosis. We hypothesized that the levels of selected biomarkers indicating endothelial injury or dysfunction, inflammation, cell apoptosis, nerve injury with neuroprotection would be different in patients and in controls. Since the profile of biomarkers may differ according to the clinical expression of vascular or neural manifestations, we also aimed to do sub-group analyses.

## Results

Descriptive characteristics of the patients and controls are presented in Table 1. Serum levels of ICAM-1, MCP-1, TM and HSP27 were found to be elevated in patients with a vibration injury compared to controls (Table 2). Difference in serum levels of TM remained almost statistically significant when including only men (*p* = 0.07), and when including only individuals without previous frostbite (*p* = 0.07, not in table). All other differences remained after sensitivity analyses.

**Table 1:** Descriptive characteristics of the 92 patients with hand-arm vibration injury, with and without Raynaud’s phenomenon (RP) and 51 controls.

	Patients			Controls
	All (n = 92)	With RP (n = 45)	Without RP (n = 47)	n = 51
Age (years)	45 (21–64)	45 (24–64)	45 (21–64)	42 (26–62)
Females	6 (7)	1 (2)	5 (11)	9 (18)
Ongoing cigarette smoking	14 (15)	8 (18)	6 (13)	2 (4)
Other medical conditions				
Previous frostbite	10^7^ (11)	6^4^ (13)	4^3^ (9)	3^1^ (6)
Cardiovascular disease	18 (20)	10 (22)	8 (17)	7(14)
Diabetes	7(8)	5 (11)	2 (4)	2 (4)
Thyroid diseases	5 (5)	4 (9)	1 (2)	1 (2)
Rheumatic disease	0 (0)	0 (0)	0 (0)	0 (0)
Polyneuropathy	4 (4)	4 (9)	0(0)	–^51^
ADHD or migraine medication	4 (4)	2 (4)	2(4)	1 (2)
Symptoms^a^				
White fingers when exposed to cold or damp (RP)	45 (49)	100 (100)	0 (0)	5 (10)
Numbness/tingling	90 (98)	45 (100)	45 (96)	7 (14)
Duration of RP (years)	5 (1–25)^57^	5 (1–25)^10^	4.5 (4–25)^47^	–^51^
Duration of numbness (years)	3 (0.2–25)^7^	4 (1–25)^2^	2 (0.2–25)^5^	–^51^
Pain/discomfort in fingers/hands when exposed to cold	80 (87)	44 (98)	36 (77)	6 (12)
Poor fine motor skills	65 (71)	36 (80)	29 (62)	4 (8)
Poor grip strength	72 (78)	36 (80)	36 (77)	4 (8)
Clinical finding^a^				
Impaired perception of touch^b^	45 (49)	29 (64)	16 (34)	6 (12)

Data presented as median (range) or n (%).

^1,2,3,4,5,7,10,47,51,57^denote the number of participants with missing data. The controls were not asked about polyneuropathy or duration of numbness or RP.

^a^Left and/or right hand.

^b^Unable to detect Semmes–Weinstein Monofilament No, 3.61, corresponding to a force of 0.4 g.

**Table 2:** Serum levels of biomarkers^a^ in 92 patients with vibration injury and 51 controls.

Biomarker	Patients (n = 92)	Controls (n = 51)	*P*-value^b^
ICAM-1 (ng/ml)	170 (130–290)	140 (80–180)	** < 0.001**
MCP-1 (pg/ml)	38 (10–100)	34 (13–73)	**0.02**
TM (ng/ml)	5.5 (2.3–39)	4.4 (0.31–34)	**0.02**
vWF (µg/ml)	16 (6.2–34)	17 (6.6–31)	0.81
VEGF (pg/ml)	< LOD (< LOD–1500)	< LOD (< LOD– < LOD)	0.19
b-FGF (pg/ml)	< LOD (< LOD–18)	< LOD (< LOD–4.8)	0.42
HGF (pg/ml)	180 (10–390)	180 (59–470)	0.64
MMP-1 (ng/ml)	3.3 (0.60–29)	3.0 (0.46–9.8)	0.77
Et-1 (pg/ml)	17 (< LOD–1800)	< LOD (< LOD–660)	0.37
CGRP (pg/ml)	< LOD (< LOD–3700)	50 (< LOD–3600)	0.93
TRPA-1 (ng/ml)	< LOD (< LOD–2.9)	< LOD (< LOD–2.4)	0.68
HSP27 (ng/ml)	3.2 (0.70–45)	2.0 (0.10–290)	** < 0.001**
GFAP (pg/ml)	< LOD (< LOD–3100)	< LOD (< LOD–2800)	0.51
MBP (ng/ml)	< LOD (< LOD–410)	< LOD (< LOD–100)	0.79
Caspase-3 (ng/ml)	1.8 (0.63–85)	2.1 (0.66–330)	0.17
Caspase-8 (ng/ml)	< LOD (< LOD–1.7)	< LOD (< LOD–7.2)	0.95
Galanin (ng/ml)	7.1 (0.7–36)	6.8 (0.6–25)	0.90

^a^Intercellular adhesion molecule 1 (ICAM-1) limit of detection (LOD), 0.002 ng/ml; monocyte chemoattractant protein 1 (MCP-1), LOD 0.6 pg/ml; thrombomodulin (TM), LOD 0.63 ng/ml; von Willebrand factor (vWf) LOD, 0.025 µg/ml; vascular endothelial growth factor (VEGF) LOD, 23 pg/ml; basic fibroblast growth factor basic (b-FGF) LOD, 2 pg/ml; hepatocyte growth factor (HGF), LOD 20 pg/ml; matrix metalloproteinase 1 (MMP-1), LOD 45 ng/ml; endothelin 1 (Et-1), LOD 10 pg/ml; calcitonin gene related peptide (CGRP), LOD 15 pg/ml; transient receptor potential ankyrin 1 (TRPA-1), LOD 0.625 ng/ml; heat shock protein 27 (HSP-27), LOD 0.21 ng/ml; glial fibrillary acidic protein (GFAP), LOD 31 pg/ml; myelin basic protein (MBP), LOD 0.16 ng/ml; caspase-3, LOD 0.041 ng/ml; caspase-8 LOD, 0.08 ng/ml; galanin LOD, 16 ng/ml.

Levels < LOD were assigned a value of half the LOD in the statistical analyses.

^b^Mann-Whitney U test used for comparison of distributions between groups. *P*-values in boldface denote statistically significant differences.

When comparing patients with RP to patients without RP; TM, vWF, CGRP, HSP27 and caspase-3 were elevated in the former (Table 3). The difference in TM remained almost statistically significant when including only individuals without previous frostbite (*p* = 0.06, not in table). However, when excluding participants with concurrent diseases, the difference for HSP27 was no longer statically significant (*p* = 0.57, not in table).

**Table 3:** Serum levels of biomarkers^a^ in patients with and without Raynaud’s Phenomenon (RP).

	Patients with RP (n = 45)	Patients without RP (n = 47)	*P*-values^b^
ICAM-1 (ng/ml)	170 (130–240)	170 (130–290)	0.58
MCP-1(pg/ml)	37 (12–95)	39 (10–104)	0.69
TM (ng/ml)	6.1 (2.7–30)	5.2 (2.3–39)	**0.01**
vWF (µg/ml)	18 (6.2–33)	14 (6.9–34)	**0.01**
VEGF (pg/ml)	< LOD (< LOD–1500)	< LOD (< LOD–510)	0.53
b-FGF (pg/ml)	< LOD (LOD–13)	< LOD (< LOD–18)	0.81
HGF (pg/ml)	180 (10–390)	180 (67–290)	0.11
MMP-1 (ng/ml)	3.7 (0.63–29)	2.9 (0.60–11)	0.09
ET-1 (pg/ml)	37 (< LOD–1800)	< LOD (< LOD–1700)	0.23
CGRP (pg/ml)	94 (< LOD–3700)	< LOD (< LOD–1400)	** < 0.001**
TRPA-1(ng/ml)	< LOD (< LOD– < LOD)	< LOD (< LOD–2.9)	0.33
HSP-27 (ng/ml)	3.4 (1.3–16)	2.8 (0.70–45)	**0.02**
GFAP (pg/ml)	< LOD (< LOD–3100)	< LOD (< LOD–2500)	0.28
MBP (ng/ml)	< LOD (< LOD–410)	< LOD (< LOD–180)	0.09
Caspase-3 (ng/ml)	2.0 (1.0–61)	1.6 (0.63–85)	**0.01**
Caspase-8 (ng/ml)	< LOD (< LOD–1.7)	< LOD (< LOD– < LOD)	0.15
Galanin (ng/ml)	7.4 (0.73–36)	6.5 (0.67–19)	0.23

^a^Intercellular adhesion molecule 1 (ICAM-1) limit of detection (LOD), 0.002 ng/ml; monocyte chemoattractant protein 1 (MCP-1), LOD 0.6 pg/ml; thrombomodulin (TM), LOD 0.63 ng/ml; von Willebrand factor (vWf) LOD, 0.025 µg/ml; vascular endothelial growth factor (VEGF) LOD, 23 pg/ml; basic fibroblast growth factor basic (b-FGF) LOD, 2 pg/ml; hepatocyte growth factor (HGF), LOD 20 pg/ml; matrix metalloproteinase 1 (MMP-1), LOD 45 ng/ml; endothelin 1 (Et-1), LOD 10 pg/ml; calcitonin gene related peptide (CGRP), LOD 15 pg/ml; transient receptor potential ankyrin 1 (TRPA-1), LOD 0.625 ng/ml; heat shock protein 27 (HSP-27), LOD 0.21 ng/ml; glial fibrillary acidic protein (GFAP), LOD 31 pg/ml; myelin basic protein (MBP), LOD 0.16 ng/ml; caspase-3, LOD 0.041 ng/ml; caspase-8 LOD, 0.08 ng/ml; galanin LOD, 16 ng/ml.

Levels < LOD were assigned a value of half the LOD in the statistical analyses.

^b^Mann-Whitney U test used for comparison of distributions between groups. *P*-values in boldface denote statistically significant differences.

Patients without RP, compared to 45 controls without RP, showed elevated serum levels of ICAM-1, MCP-1 and HSP27 (Table 4). When including only individuals without previous frostbites the *p*-value for MCP-1 was 0.06, i.e., almost statistically significant. Caspase-3 was lower in patients than in controls, but this difference disappeared when including only participants without concurrent disease (*p* = 0.25, not in table). All other results remained statistically significant according to the sensitivity analyses.

**Table 4:** Serum levels of biomarkers^a^ in patients without Raynaud’s phenomenon (RP) compared to controls without RP.

	Patients without RP (n = 47)	Controls without RP^c^ (n = 46)	*P*-values^b^
ICAM-1 (ng/ml)	170 (130–290)	140 (80–180)	** < 0.001**
MCP-1 (pg/ml)	39 (10–100)	34 (13–68)	**0.02**
TM (ng/ml)	5.2 (2.3–39)	4.4 (0.31–28)	0.39
vWF (µg/ml)	14 (6.9–34)	16 (6.6–31)	0.11
VEGF (pg/ml)	< LOD (< LOD–510)	< LOD (< LOD– < LOD)	0.32
b-FGF (pg/ml)	< LOD (< LOD–18)	< LOD (< LOD–3.7)	0.28
HGF (pg/ml)	180 (67–290)	190 (59–470)	0.24
MMP-1 (ng/ml)	2.9 (0.6–11)	3.0 (1.0–9.8)	0.43
Et-1 (pg/ml)	< LOD (< LOD–1700)	< LOD (< LOD–580)	0.80
CGRP (pg/ml)	< LOD (< LOD–1400)	29 (< LOD–3600)	0.14
TRPA-1 (ng/ml)	< LOD (< LOD–2.9)	< LOD (< LOD–2.4)	1.0
HSP27 (ng/ml)	2.8 (0.70–45)	1.8 (< LOD–290)	** < 0.001**
GFAP (pg/ml)	< LOD (< LOD–2500)	< LOD (< LOD–2800)	0.66
MBP (ng/ml)	< LOD (< LOD–180)	< LOD (< LOD–0.95)	0.46
Caspase-3 (ng/ml)	1.6 (0.63–85)	2.0 (0.66–330)	**0.02**
Caspase-8 (ng/ml)	< LOD (< LOD– < LOD)	< LOD (< LOD–7.2)	0.31
Galanin (ng/ml)	6.5 (0.67–19)	5.9 (0.57–25)	0.96

^a^Intercellular adhesion molecule 1 (ICAM-1) limit of detection (LOD), 0.002 ng/ml; monocyte chemoattractant protein 1 (MCP-1), LOD 0.6 pg/ml; thrombomodulin (TM), LOD 0.63 ng/ml; von Willebrand factor (vWf) LOD, 0.025 µg/ml; vascular endothelial growth factor (VEGF) LOD, 23 pg/ml; basic fibroblast growth factor basic (b-FGF) LOD, 2 pg/ml; hepatocyte growth factor (HGF), LOD 20 pg/ml; matrix metalloproteinase 1 (MMP-1), LOD 45 ng/ml; endothelin 1 (Et-1), LOD 10 pg/ml; calcitonin gene related peptide (CGRP), LOD 15 pg/ml; transient receptor potential ankyrin 1 (TRPA-1), LOD 0.625 ng/ml; heat shock protein 27 (HSP-27), LOD 0.21 ng/ml; glial fibrillary acidic protein (GFAP), LOD 31 pg/ml; myelin basic protein (MBP), LOD 0.16 ng/ml; caspase-3, LOD 0.041 ng/ml; caspase-8 LOD, 0.08 ng/ml; galanin LOD, 16 ng/ml.

Levels < LOD were assigned a value of half the LOD in the statistical analyses.

^b^Mann-Whitney U test used for comparison of distributions between groups. *P*-values in boldface denote statistically significant differences.

^c^Five individuals in the control group had RP and were excluded.

Serum levels of VEGF, b-FGF, HGF, MMP-1 and caspase-8, GFAP, MBP and galanin showed no differences among the studied groups. Serum levels of MMP-12, β-NGF and NT-3 were below the detection limits in all samples.

## Discussion

**Figure 1:**
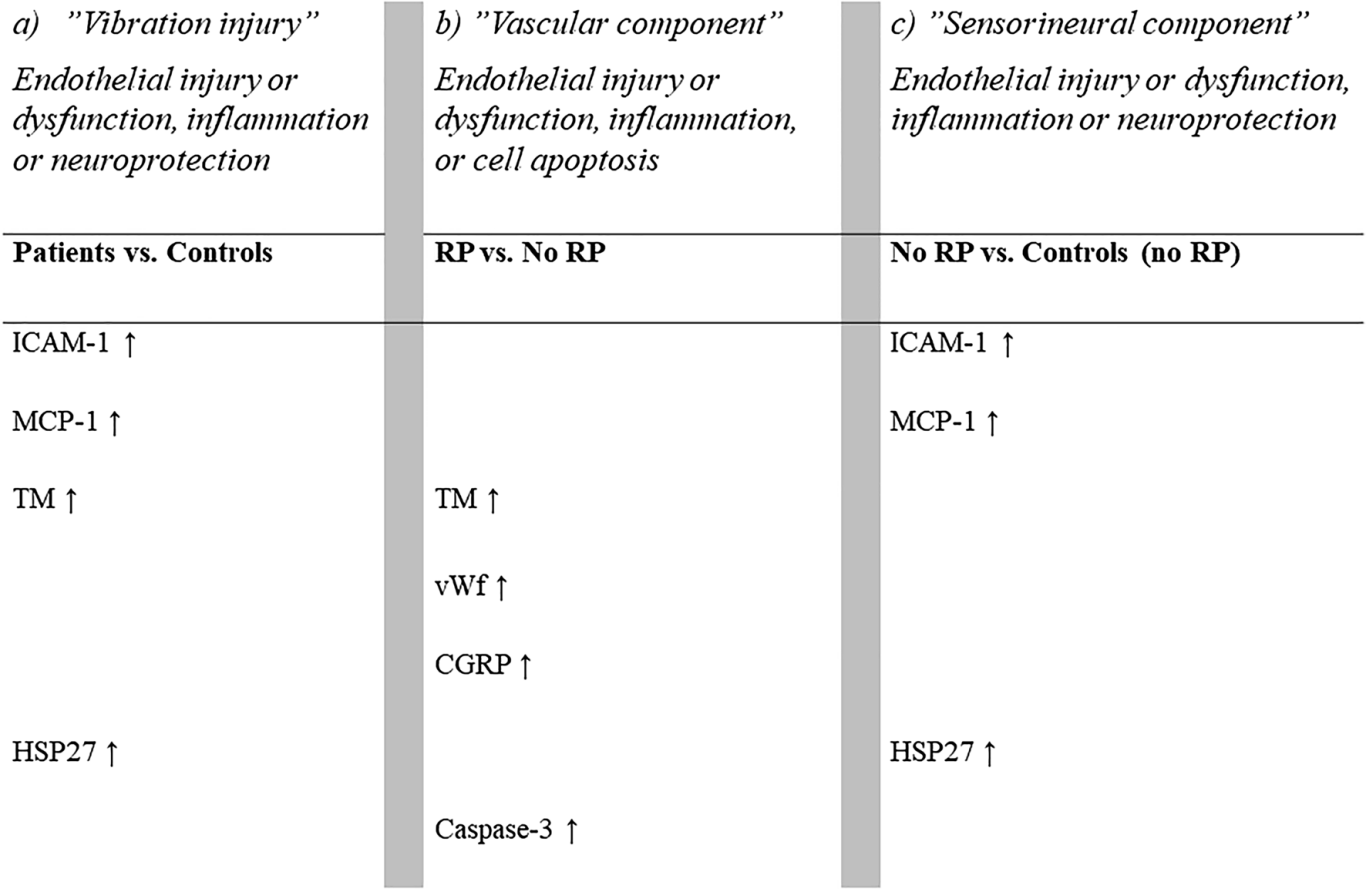
Schematic illustration of elevated serum levels of biomarkers (after sensitivity analyses) for: (**a**) patients vs. controls; (**b**) patients with Raynaud’s phenomenon (RP) vs. patients without RP; (**c**) patients without RP vs. controls without RP.

Patients with hand-arm vibration injury had elevated serum levels of biomarkers related to endothelial injury or dysfunction, inflammation (TM, ICAM-1 and MCP-1), and to neuroprotection in relation to nerve fibre injury (HSP27) compared to controls (Fig. 1a). Comparing patients with and without RP revealed a different pattern, with elevated levels of TM, vWf, CGRP and caspase-3, indicating a higher degree of endothelial injury, endothelial dysfunction, inflammation, and cell apoptosis among patients with the vascular component of vibration injury than among those without RP (Fig. 1b). When comparing patients with only the sensorineural component of injury (without RP) to controls (without RP), elevated levels of ICAM-1, MCP-1 and HSP27 were shown (Fig. 1c).

The elevated levels of ICAM-1 in patients compared with controls are in line with a previous study^19^, however, the elevated levels of MCP-1 in patients with vibration injuries is a novel finding^18,41^. Upregulation of ICAM-1 has been shown in response to both low and high amplitude of cyclic strain applied^14,42^. The elevated levels of ICAM-1 and MCP-1 suggests an inflammatory response in vibration injuries. No difference in serum levels between patients with and without RP suggests that these biomarkers are not primarily linked to the clinical expression of RP, but rather the “sensorineural component” of injury (Fig. 1).

The elevated levels of TM found in patients with vibration injuries compared with controls is in accordance with previous findings^9,19,43^. Since TM was elevated in patients with RP, but not in the patient group with only the sensorineural component of injury, it appears that TM is involved in the clinical presentation of RP. Under normal conditions, TM is present in the blood at low concentrations, but it is elevated in several pathological conditions associated with endothelial dysfunction, such as cardiovascular, inflammatory, infectious and metabolic diseases^44^. Although patients with RP reported higher prevalence of e.g., cardiovascular diseases and current smoking, the results remained after sensitivity analyses.

Patients with RP showed elevated levels of vWf and CGRP, both of which are associated with primary and secondary forms of RP^22,31,45–47^. Increased levels of vWf have previously been associated with inflammation and intimal hyperplasia^48^; thus, such mechanisms may be at play in these patients. In conditions with high shear stress, it has been shown that globular-shaped vWF unfolds into long-chain structure, making the adherence of platelets to the injured endothelial surface easy and enhancing further leukocyte recruitment^48^. At low shear stress, on the other hand, with intact endothelium, smooth muscle cell proliferation occurs without platelet activation and the degree of intimal hyperplasia is proportional to the expression of vWF^48^. Structural injury with increased smooth muscle thickness has been shown as result of vibration injury in animal models^2,20,49^.

CGRP is a potent vasodilator released from perivascular sensory nerve endings of unmyelinated C-fibres and myelinated thin Aδ-fibres^50^. On administration of CGRP antagonists for migraine treatment, subjects have reported new onset of RP^51^. Interestingly, in this study patients with RP showed elevated, instead of decreased levels, which is in line with a previous study on patients with scleroderma^52^. However, a reduction of CGRP-staining nerves in histopathological examination of biopsies from patients with primary RP, secondary RP due to scleroderma, and in patients with vibration-induced RP has been shown^22,31,46^. Also, lower serum levels of CGRP were shown in patients with long standing scleroderma, possibly due to chronic inflammation-induced depletion of CGRP^50^. A plausible explanation for the present elevated levels of CGRP, could be an increased CGRP-release as a vaso- or neuroprotective mechanism. CGRP has been shown to inhibit intima hyperplasia and expression of the inflammatory marker MCP-1 in chronic induced inflammation^50^. A normalised function of sensory nerves was seen on upregulation of CGRP in the dorsal root ganglion neuron on capsaicin administration in diabetic induced neuropathy in rats^53^.

Furthermore, experimentally induced activation of TRPA-1, channels, associated with cold intolerance and inflammatory pain^24,54^, showed a CGRP-induced neurogenic vasodilation^23^. A possible link with the prevalent symptom cold intolerance in patients with hand-arm vibration injury is therefore highly interesting and warrants further investigation.

Patients with RP showed higher serum levels of caspase-3 than patients without RP. This is to the best of our knowledge a novel finding. Disrupted vascular endothelial cells with vacuole formation and altered levels in myosin light chain kinase as a result of vibration induced trauma have been observed in an animal model^20^. Inhibiting myosin light chain kinase was shown to induce apoptosis in vitro and in vivo^55^. Although caspase-3 seems to be related to the clinical presentation of RP among vibration-injured patients, its exact origin from vascular or cells related to nerve fibres, e.g., Schwann cells, cannot completely be revealed.

Finally, in contrast with a previous study, where no elevated levels of HSP27 were shown in hand-arm vibration exposed workers^34^, HSP27 was elevated both in patients compared to controls, and in patients with only the sensorineural component of injury compared to controls. This indicates that compensatory neuroprotective mechanisms, such as upregulation of HSPs to preserve function, as seen in subjects with diabetes neuropathy^36,56^, are present in patients with hand-arm vibration injury^57,58^ despite no increased levels of GFAP as a sign of nerve injury. In fact, similar structural changes in nerve biopsies from the posterior interosseous nerve, i.e., nerve fibre degeneration, demyelination and fibrosis, as seen in subjects with diabetes neuropathy have been shown in patients with hand-arm vibration injury^26^.

In this study we have looked at biomarkers associated the vascular and neurosensory components of injury including markers indicating remodelling of the extracellular matrix (growth factors, MMP-1 and MMP-12). There are yet other interesting biomarkers that could reflect the site of injury, i.e., bone and cartilage metabolism. Today there is a suspicion, but not sufficient scientific evidence concerning an association between vibration exposure and osteoarthritis in the hands^59^.A strength of this study is the large number of patients in whom hand-arm vibration injury had been ascertained by a specialist in OEM. Also, a vast number of biomarkers with a theoretically hypothesis driven approach of plausible pathophysiological mechanisms were assessed. The control group was smaller and due to practical reasons, had a lower proportion of smokers. To assess confounding, we performed sensitivity analyses on subgroups without smokers, as well as other factors that may have influenced the results. A limitation of this study is the way used to assess some known risk factors for RP, e.g., previous frostbite^60^. In this study, information on frostbite was based on answering yes or no to a single question, with no further assessment, and many failed to answer this question, which could have biased the results. Differences in blood biomarker concentrations due to diurnal variations were avoided by collecting all blood samples before noon. A limitation of the study is that blood samples were not collected from patients and controls during the same time-period. The recruitment of the controls was delayed due to the Covid-19 pandemic, and the samples from the controls were collected two years later than those in the patient group. Samples were stored at  − 80 °C, but the concentrations of biomarkers may potentially be biased towards higher biomarker concentrations in samples from controls, depending on the stability of the biomarkers, due to a shorter storage time However, for the differences presented in this study, the serum levels of biomarkers were more elevated in patients than controls. Despite this, the difference in storage time before analysis could add to the uncertainty of the results.

## Conclusions

Patients with hand-arm vibration injuries showed elevated serum levels of biomarkers related to endothelial injury or dysfunction, inflammation (ICAM-1, MCP-1, TM), and neuroprotection in relation till nerve injury (HSP27) compared to controls. In addition, patients with RP showed elevated levels of biomarkers associated with intima hyperplasia (vWf) and possibly vaso- or neuroprotective compensatory (CGRP and caspase-3), or apoptotic mechanisms (caspase-3). This study adds important knowledge on pathophysiological mechanisms that can contribute to implementation of a more objective method for diagnosis of hand-arm vibration injury.

## Methods

### Study design

This study has an observational case–control design. Cases were patients with hand-arm vibration injury. Blood samples were collected from patients diagnosed with hand-arm vibration injury by a specialist in occupational and environmental medicine (OEM), in conjunction with their visit to an OEM outpatient clinic in southern Sweden. The patients filled in a questionnaire before their visit, and then underwent a clinical examination. They were instructed not to use vibrating tools during the twelve hours before attending the clinic, and not to use nicotine in any form one hour before their visit, and to keep their hands warm. Controls without a known diagnosis of hand-arm vibration injury, and without ongoing exposure to vibration at work were enrolled and examined at their workplaces. They filled in a short version of the questionnaire and were examined regarding their perception of touch.

### Study group

Ninety-two patients (86 men and 6 women) were enrolled from August 2018 until February 2020. Eight of them were no longer exposed to hand-arm vibration at the time of the study. Their characteristics have been described in a previous study^61^. One participant claimed not to have diabetes, but were on diabetes medication and had elevated HbA1c, and was thus classified as having diabetes in the present study.

Controls were enrolled from five different workplaces from March 2022 to October 2022. All the employees at these workplaces were invited to participate, and the exclusion criteria were having a diagnosis of vibration injury or ongoing exposure to vibration at work. The controls worked with logistics, as warehouse workers, janitors, chefs, and office workers. Fifty-three individuals were recruited as controls, but two men were excluded since they had been exposed to vibration previously, reported tingling/numbness, or showed impaired perception of touch, i.e., a hand-arm vibration injury could not be ruled out. The control group thus consisted of 44 men and 9 women. As the blood samples collected from the controls were also used in another study on exposure to chromium at work, they were required not to be heavy smokers.

### Symptoms

The part of the questionnaire completed by both the patients and the controls included six questions on symptoms, where the responses (Yes) or (No) were indicated separately for the right and left hand: Do you experience: (a) numbness or tingling?, (b) numbness or tingling during the night?, (c) pain/discomfort in fingers/hands during cold exposure?, (d) white fingers when exposed to cold or dampness?, (e) poor grip strength?, (f) poor fine motor skills or clumsiness?. The patients were also asked about the duration of symptoms of RP and numbness and tingling.

### Perception of touch

The patients were examined with Semmes–Weinstein monofilaments according to a previously described protocol^61^. The controls were only tested with filament No. 3.61 (corresponding to 0.4 g force). Those who could not detect filament stimulation with filament No. 3.61, were considered to have impaired perception of touch.

### Biomarkers

Blood samples were collected after clinical examination, before noon, in 7 ml serum separation tubes with gel. After 30 min, serum was removed by centrifugation at 2000 × *g* for 10 min, and the samples were stored at  − 80 °C until analysed. Serum concentrations of TM, vWf, ET-1, GFAP, HSP27, CGRP, MBP and TRPA-1 were determined using commercially available ELISA kits, following the instructions provided by the manufacturers (TM from BioVendor, Brno, Czech Republic; vWf from Abnova, Taipei City, Taiwan; ET-1 and caspase-3 from RayBiotech, Norcross, GA, USA; GFAP from Proteintech, Manchester, UK; HSP27 from Millipore, Saint Louis, MO, USA; CGRP, MBP, caspase-8 and galanin from FineTest, Hubei, China and TRPA-1 from Cloud-Clone Corp., Katy, TX, USA). All samples were diluted 1:2 except HSP27 and caspase-3 (1:3 dilution), galanin (1:10 dilution) and vWf (1:100 dilution), and samples with levels exceeding the highest calibration point were also further diluted.

Serum concentrations of ICAM-1 (1:100 dilution), MCP-1 and VEGF (1:4 dilution) were determined using multiplexed immunoassays from Bio-Rad (Hercules, CA, USA), while serum concentrations of MMP-1, MMP-12, b-FGF, β-nerve growth factor (β-NGF), HGF and neurotrophin (NT)-3 (1:2 dilution) were analysed using multiplexed Luminex discovery assays from Bio-Techne (Minneapolis, MN, USA). The multiplexed assays were prepared and analysed on a Luminex platform (Bio-Plex 200, Bio-Rad, Hercules, CA, USA) according to the manufacturer’s instructions. All samples were analysed in duplicate. Samples with values below the limit of detection (LOD) were assigned a value of half the LOD in the statistical analyses.

### Data handling and statistical analyses

SPSS IBM Statistics for Windows, Version 25.0 (IBM Corp., Armonk, NY, USA) was used for all statistical analysis. Data on serum levels of biomarkers were evaluated and found not to be normally distributed. The results are therefore presented as medians with ranges. Mann–Whitney U tests were used to compare serum levels in patients and controls, in patients with and without RP, and in patients who exhibited only the sensorineural component of injury (i.e., no RP) and controls without RP (five controls with RP were excluded from this comparison). Male sex, smoking, previous frostbite, and concurrent disease (cardiovascular disease, diabetes, thyroid disease, polyneuropathy or pharmacologically treated ADHD or migraine) were more common among the patients than among the controls, and in patients with, than without, RP. Therefore, sensitivity analyses were performed. The Mann–Whitney U-test was repeated in subgroups including only men, only non-smokers, only individuals without previous frostbite, and finally only individuals without concurrent disease. A *p*-value below 0.05 was considered to indicate that there was a statistically significant difference between two groups.

### Informed consent

Participants gave their informed consent to participate in the study before taking part.

### Ethics approval

The study was conducted in accordance with the Declaration of Helsinki, and approved by the Regional Ethics Board in Lund, Sweden (No. 2018/15).

## Data Availability

The datasets presented in this article are not readily available as public access to data is restricted to Swedish Authorities (Public Access to Information and Secrecy Act), but data can be made available for researchers after a special review that includes approval of the research project by both an Ethics Committee and the Authorities’ Data Safety Committees. Contact the corresponding author for request.

## Abbreviations

RP: Raynaud’s Phenomenon
TM: Thrombomodulin
vWF: von Willebrand factor
ICAM-1: Intercellular adhesion molecule 1
MCP-1: Monocyte chemoattractant protein 1
CGRP: Calcitonin gene-related peptide
HSP27: Heat shock protein 27
Et-1: Endothelin 1
MMP-1: Matrix metalloproteinase 1
MMP-12: Metalloproteinase 12
HGF: Hepatocyte growth factor
b-FGF: Basic fibroblast growth factor
VEGF: Vascular endothelial growth factor
MBP: Myelin basic protein
GFAP: Glial fibrillary acidic protein
TRPA-1: Transient receptor potential ankyrin 1
GAL: Galanin
β-NGF: β-Nerve growth factor
NT-3: Neurotrophin
LOD: Limit of detection
